# Case Report: a novel homozygous *ASNS* variant in a Chinese female with severe microcephaly, encephalopathy and epilepsy

**DOI:** 10.3389/fnins.2025.1570160

**Published:** 2025-05-12

**Authors:** Shuangxi Cheng, Fang Zhang, Qingming Wang, Jianfei Zhang, Guizhen Lyu, Yanwei Li, Xinlong Zhou, Haiming Yuan

**Affiliations:** ^1^Dongguan Maternal and Child Health Care Hospital, Dongguan, China; ^2^Dongguan Labway Clinical Laboratory Co., Ltd., Dongguan, China

**Keywords:** *ASNS*, microcephaly, psychomotor delay, epilepsy, brain anomalies

## Abstract

Asparagine synthetase deficiency (ASNSD; OMIM# 615574) is a severe autosomal recessive neurodevelopmental disorder caused by biallelic pathogenic variants in *ASNS* (OMIM# 108370). Clinical features of ASNSD include congenital microcephaly, profound psychomotor impairment, progressive encephalopathy, refractory epilepsy, and characteristic neuroimaging abnormalities. Since its initial description, approximately 100 cases have been documented worldwide with 60 distinct *ASNS* variants reported. Here, we report a Chinese patient with prenatal microcephaly, intrauterine growth retardation (IUGR) and reduced middle cerebral artery blood flow velocity. Postnatally, she presented with progressive microcephaly, profound psychomotor delay and intractable epilepsy. Brain MRI showed corpus callosum hypoplasia, cerebellar hypoplasia, delayed myelination, cortical atrophy, enlarged ventricles and gyral simplification. Whole-exome sequencing (WES) was applied to detect the causative variants and identified a novel homozygous variant c.4 T > G (p.Cys2Gly), in *ASNS* in our patient that was inherited from the heterozygous unaffected parents. Our report contributes to the expanding genotypic and prenatal phenotype spectrum of ASNSD.

## Introduction

Asparagine synthetase deficiency (ASNSD, MIM# 615574) is a rare autosomal recessive neurologic disorder, characterized by congenital microcephaly, progressive encephalopathy, severely delayed psychomotor development, intractable epilepsy, feeding difficulties and dystonia. Brain anomalies include cortical atrophy, cerebellar hypoplasia, delayed myelination, enlarged ventricles and thin corpus callosum. The disease may show onset in utero or at birth and may cause death usually in infancy. ASNSD is caused by biallelic pathogenic variants in *ASNS* (Ruzzo et al., 2013; Sacharow et al., 2018). The *ASNS* gene (MIM# 108370) consists of 13 exons and encodes 561-amino acids asparagine synthetase, which catalyzes the transfer of ammonia from glutamine to aspartic acid to form asparagine. The ASNS protein encompasses two functional domains: glutamine amidotransferase type-2 (Cys2-Lys191) and asparagine synthetase domains (His213-Tyr536), and is highly expressed in both the developing embryonic and adult brain (Lomelino et al., 2017). Pathogenic variants in *ASNS* cause defective asparagine synthesis, which leads to asparagine deficiency or aspartate/glutamate accumulation in the brain, thus causes serious neurological diseases (Ruzzo et al., 2013).

Currently, approximately 100 individuals with ASNSD have been described, and 60 pathogenic variants in *ASNS* have been identified (Ruzzo et al., 2013; Yamamoto et al., 2017; Galada et al., 2018; Schleinitz et al., 2018; Costa et al., 2019; Kahrizi et al., 2019; Shaheen et al., 2019; Akesson et al., 2020; Alharby et al., 2020; van der Ven et al., 2021; Staklinski et al., 2022; Jahanpanah et al., 2024; Song et al., 2024). Thus, it is required to further enrich the clinical characteristics of this disorder and expand the mutation spectrum of *ASNS*. In this study, we identified a novel homozygous variant c.4 T > G (p.Cys2Gly) in *ASNS*, in a 6-month-old Chinese female who displayed novel prenatal phenotypes and typical postnatal features of ASNSD. This study expands the mutation spectrum of *ASNS* and enriches the clinical features of this disorder.

## Materials and methods

### Ethical compliance

This study was authorized by the Ethics Committee of Dongguan Maternal and Child Health Care Hospital (DMCH 202307). Written informed consent was obtained from the parents of the patient for the release of any potentially identifiable image or data contained in this paper.

### Whole exome sequencing (WES) and sanger sequencing

Genomic DNA was extracted using a DNA extraction kit (Qiagen) according to the manufacturer’s instructions. WES was performed to screen for genetic variants in this patient. Sequencing was operated with an Illumina NovaSeq 6,000 (Illumina, San Diego, CA, United States). The bcl2fastq2 Conversion Software (v2.20) was used for extracting Fastq files, and all reads were mapped to the human genome (GRCh37/hg19) by using BWA (v0.2.10) with default parameters. The Genome Analysis Toolkit (GATK; v.3.7) HaplotypeCaller was used for detecting variants. The aligned reads were visualized using the Integrated Genome Viewer (IGV). Common variants were filtered based on their frequencies in the Genome Aggregation Database (gnomAD)^1^ and our internal database. The suspected variant was confirmed by Sanger sequencing. The pathogenicity of the sequence variants was assessed according to ACMG/AMP guidelines (Richards et al., 2015).

## Results

### Clinical report

The proband was a 6-month-old female infant born to a nonconsanguineous Chinese couple. The family history was unremarkable, with a healthy elder sibling. Microcephaly and cleft palate were observed by ultrasonography at a gestational age of 24 weeks. Fetal magnetic resonance imaging (MRI) at this time showed no brain structural abnormalities. Amniocentesis revealed a normal karyotype and absence of pathogenic copy number variants by chromosomal microarray analysis. Serial ultrasonographic monitoring confirmed progressive microcephaly at 27 and 30 weeks of gestation and newly detected intrauterine growth restriction (IUGR) at 35 weeks, accompanied by diminished peak systolic velocity in the middle cerebral artery.

**Figure 1:**
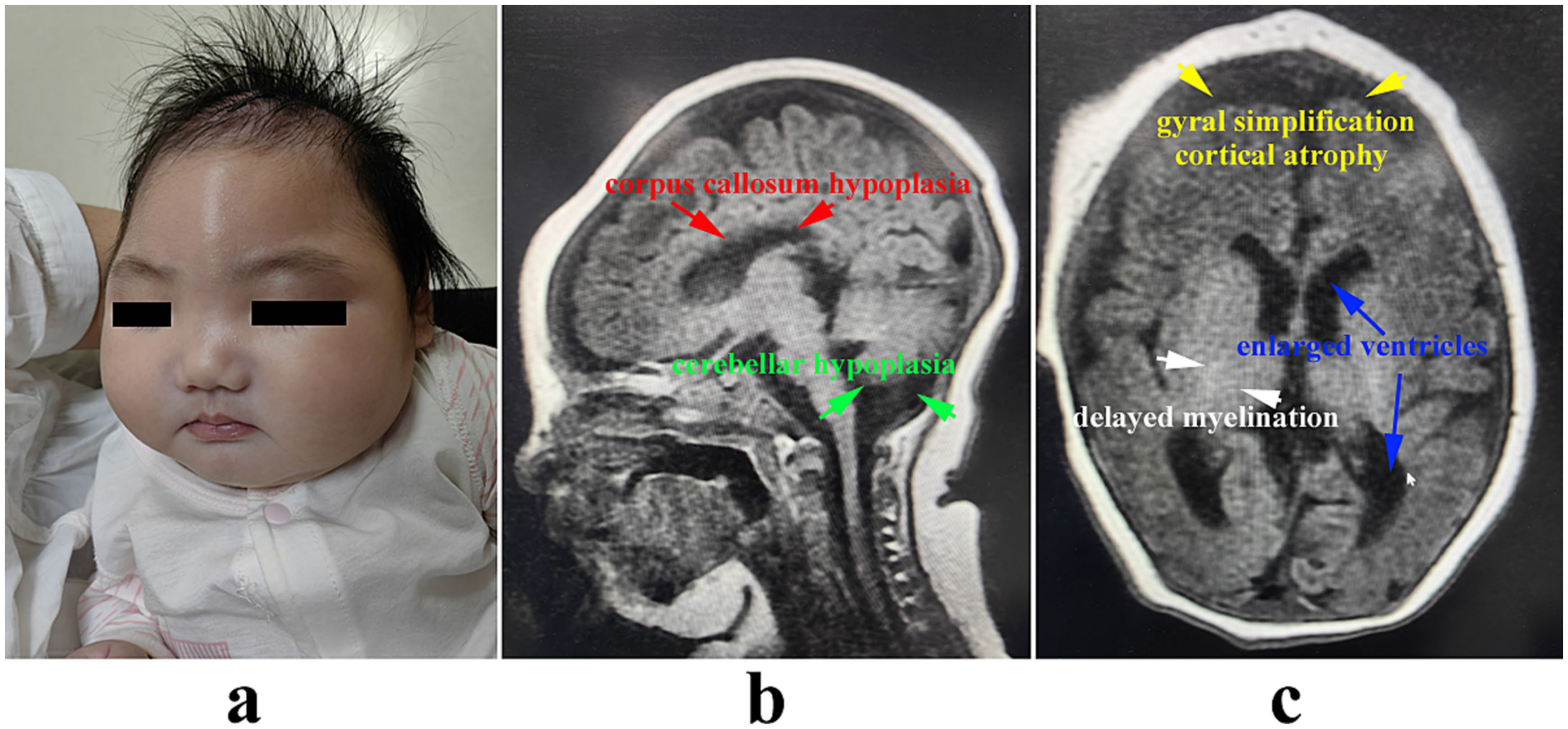
Image features of the patient. Severe microcephaly was noticed **(a)**. Brain MRI showed corpus callosum hypoplasia, cerebellar hypoplasia **(b)**, delayed myelination, cortical atrophy, enlarged ventricles and gyral simplification **(c)**.

The infant was delivered vaginally at 37 weeks with anthropometric parameters consistent with severe growth restriction: birth weight 2.4 kg (−2.5 SD), length 45.0 cm (−3.2 SD), and head circumference (HC) 30.0 cm (−3.5 SD). Postnatal clinical deterioration manifested at 3 months of age, characterized by profound global developmental delay (failure to achieve head control), hypotonia and feeding difficulties. Anthropometric measurements at this stage confirmed failure to thrive: weight 4.74 kg (−2.8 SD), length 55 cm (−2.3 SD), and HC 32 cm (<−6 SD) (Figure 1a). At 4 months, she developed intractable epilepsy, which was characterized by upturned eyes, closed teeth, clenched fists and twitching limbs. Each episode lasted for 1–5 min, with 5–10 episodes every day. Electroencephalography (EEG) showed extensive anomalies with remarkable epileptic discharge, including large amounts of multifocal low-medium-voltage sharp spikes/polyspikes-and-slow waves. Her seizures could not be effectively controlled by anti-epilepsy medicines including levetiracetam and sodium valproate. Brain MRI showed corpus callosum hypoplasia, cerebellar hypoplasia (Figure 1b), delayed myelination, cortical atrophy, enlarged ventricles and gyral simplification (Figure 1c). She did not achieve any developmental milestones and she did not make eye contact. She died at 6 months due to respiratory failure and status epilepticus.

### Genetic analysis

**Figure 2:**
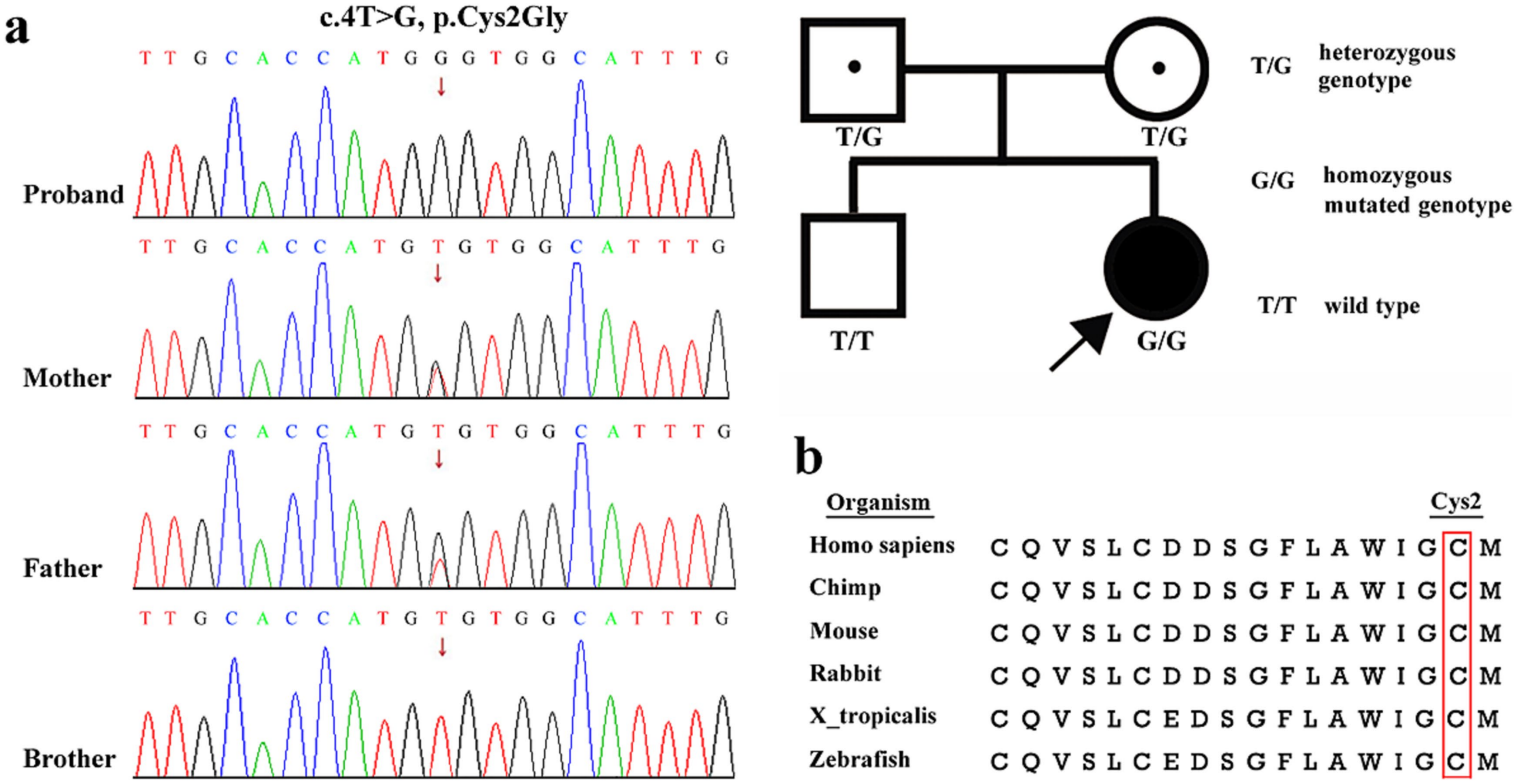
Genotypic information of the family. **(a)** Variant identification by Sanger sequencing. A homozygous variant c.4 T > G, p.Cys2Gly in *ASNS* was detected in the patient. Her asymptomatic parents were heterozygous carriers and her elder brother was wild type. The red arrow indicates the variant site. The pedigree shows segregation of the identified *ASNS* variant. **(b)** Location of patient’s *ASNS* missense variant (c.4 T > G, p.Cys2Gly) on a highly conserved multi-species alignment of ASNS protein sequence, red box indicates the residue p.Cys2.

WES revealed a novel homozygous missense variant, c.4 T > G (p.Cys2Gly), in *ASNS* in the proband. Both unaffected parents were heterozygous carriers. Her brother was wild type (Figure 2a). The variant is absent in the Genome Aggregation Database or the 1,000 Genomes Project (PM2). Evolutionary conservation analysis revealed that Cys2 residue is highly conserved among different species and is located in the glutamine amidotransferase type-2 domain (PM1) (Figure 2b). Previous studies have shown that this domain is critical for the synthesis of asparagine (Van Heeke and Schuster, 1989). The variant was predicted to have a damaging effect on the gene product by multiple in silico prediction tools (SIFT, PolyPhen-2 and MutationTaster) (PP3). Then, 3-dimensional (3D) protein modeling was performed to assess the impact of p.Cys2Gly on the stability of ASNS protein. Alphafold2 modeling and PyMOL mapping analysis showed that the p.Cys2Gly variant has no destructive effect on the tertiary structure of ASNS protein (Supplementary Figure S1). The ΔΔG (kcal/mol) value was derived from the protein stability prediction analysis, and its negative value showed that the protein stability was affected after mutation (Supplementary Figure S2). Furthermore, the patient’s manifestations were strikingly similar to those of ASNSD (PP4), and WES also excluded other possible known genetic causes. Thus, the variant was assessed as clinically likely pathogenic according to the ACMG/AMP guidelines and was responsible for clinical features of our patient.

## Discussion

Biallelic pathogenic variants in *ASNS* lead to Asparagine synthetase deficiency (ASNSD), a rare neurologic disorder. Currently, only 60 variants in *ASNS* have been revealed in approximately 100 ASNSD patients (Ruzzo et al., 2013; Yamamoto et al., 2017; Galada et al., 2018; Schleinitz et al., 2018; Costa et al., 2019; Kahrizi et al., 2019; Shaheen et al., 2019; Akesson et al., 2020; Alharby et al., 2020; van der Ven et al., 2021; Staklinski et al., 2022; Jahanpanah et al., 2024; Song et al., 2024).

In this study, we reported a 6-month-old Chinese patient who had severe psychomotor delay, progressive microcephaly, intractable epilepsy, hypotonia, feeding difficulties and brain dysplasia. The phenotypes were strikingly similar to those of ASNSD. A novel homozygous missense variant, c.4 T > G (p.Cys2Gly), in *ASNS* was identified. The variant was categorized as clinically likely pathogenic and was linked to our patient’s clinical symptoms. This novel variant further expands the *ASNS* mutation spectrum and could help to the genetic diagnosis of ASNSD.

**Figure 3:**
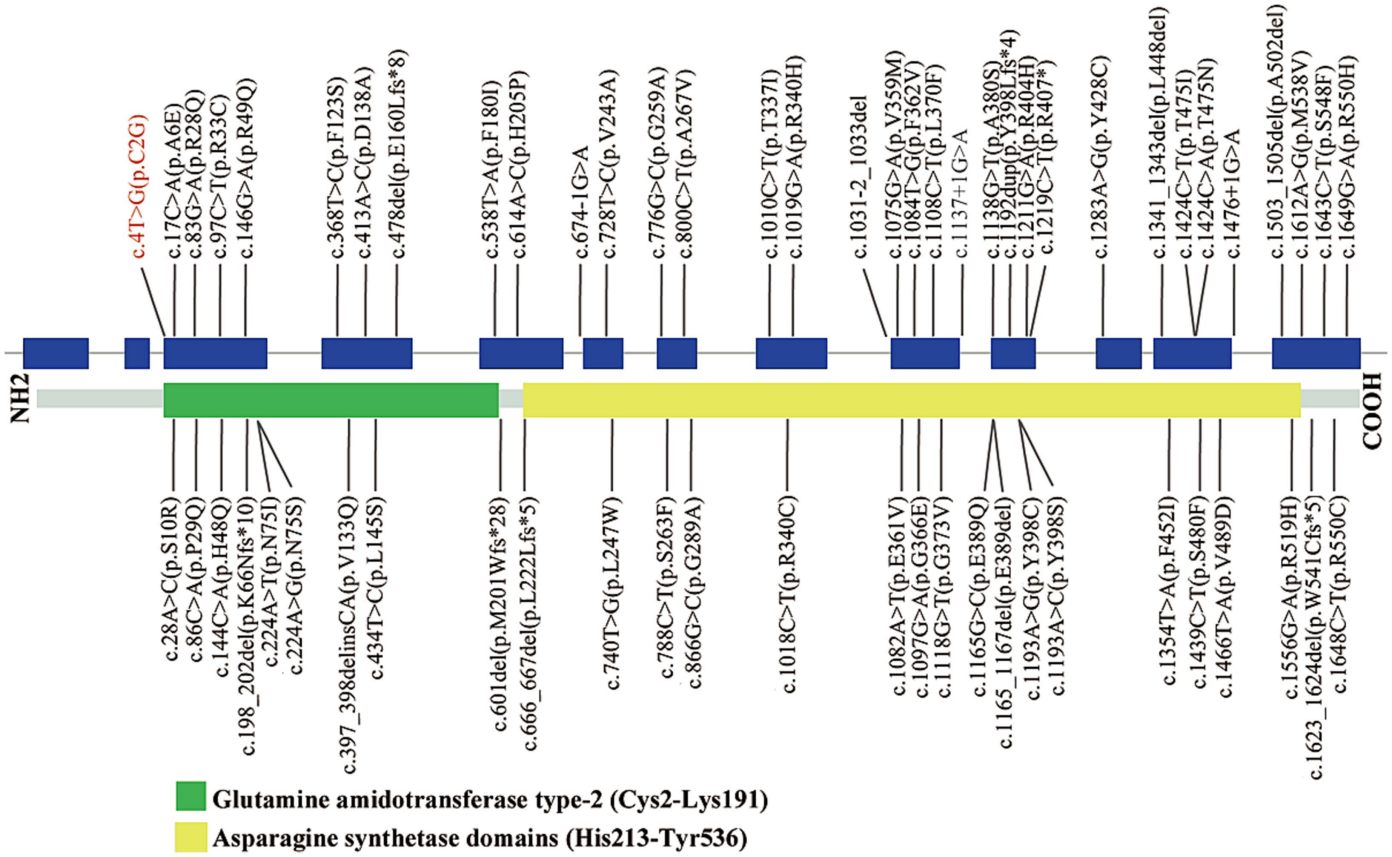
Schematic representation of *ASNS* variants identified to date. The structure of *ASNS* contained 13 exons (blue rectangles), and introns (gray horizontal line); lower side: the ASNS protein domains: glutamine amidotransferase type-2 (Cys2-Lys191) and asparagine synthetase domains (His213-Tyr536). Black: Variants identified in the literature; Red: Novel variants detected in this study.

Next, we reviewed all *ASNS* variants and clinical features in individuals with ASNSD. A total of 61 variants, including the newly identified variant here, were revealed. *ASNS* missense variants, null variants (nonsense, frameshift and splicing) and inframe variants accounted for 75.4% (46/61), 21.3% (13/61) and 3.3% (2/61), respectively. Variants were distributed across all coding exons and flanking introns (Figure 3). It was noticed that missense variants were the most common variants. Currently, only a few biallelic null variants (c.1219C > T, p.Arg407Ter; c.198_202del, p.Lys66fs; c.1137 + 1G > A; c.674-1G > A; c.1476 + 1G > A) have been detected in affected patients with early death (Alharby et al., 2020; Schleinitz et al., 2018; Akesson et al., 2020). It suggested that biallelic null variants could lead to clinical outcomes that are too severe to be compatible with live birth.

Postnatal clinical phenotypes of ASNSD have been reported relatively in detail. However, prenatal profiles of ASNSD were infrequently described. Currently, only two prenatal cases with detailed clinical information were reported. Zhu et al. (2023) reported a Chinese family with a fetus displaying microcephaly, IUGR and encephalodysplasia. Encephalodysplasia included reduced biparietal diameter and thinner white matter. The compound heterozygous variants, c.97C > T (p.Arg33Cys) and c.1031-2_1033del, in *ASNS* were identified. Churchill et al. (2020) described a Caucasian descent couple who had a 2-year son presenting typical features for ASNSD. A compound heterozygous variants, c.478del (p.Glu160Leufs*8) and c.1283A > G (p.Tyr428Cys), in *ASNS* were revealed. The couple had another pregnancy. The imaging studies of the fetus showed microcephaly, white matter volume loss, corpus callosum hypoplasia, small cerebellum and brainstem. The genetic testing confirmed that the fetus carried the same compound heterozygous variants in *ASNS*. Here, our patient displayed typical postnatal manifestations of ASNSD. Furthermore, she also presented with prenatal features including microcephaly, IUGR and a decrease in blood flow velocity in the middle cerebral artery. Decrease of blood flow velocity in the middle cerebral artery had never been reported in prenatal phenotypes of ASNSD. Our study enriched prenatal clinical characteristics of ASNSD.

In conclusion, we identified a novel homozygous variant c.4 T > G (p.Cys2Gly) in *ASNS* in a Chinese female who presented with typical postnatal symptoms of ASNSD. And prenatal profiles were also described. This study will expand the variant spectrum of *ASNS* and enrich our knowledge toward clinical characteristics, management and genetic counseling of ASNSD, which needs to be further studied.

## Data Availability

The data that support the findings of this study are available on request from the corresponding author. The data are not publicly available due to privacy or ethical restrictions.

## Footnotes

^1^ https://gnomad.broadinstitute.org/
