# Supplementary material for: Case Report: a novel homozygous ASNS variant in a Chinese female with severe microcephaly, encephalopathy and epilepsy
Source: Front Neurosci. 2025 May 12;19:1570160. doi: 10.3389/fnins.2025.1570160 (PMC12104238; doi:10.3389/fnins.2025.1570160)
Supplement: Supplementary file 1 [file Data_Sheet_1.docx]

**Case report: A novel homozygous *ASNS* variant in a Chinese female with**

**severe microcephaly, encephalopathy and epilepsy**

Shuangxi Cheng^1^, Fang Zhang^1^, Qingming Wang^1^, Jianfei Zhang^1^, Guizhen Lyu^2^, Yanwei Li^2^, Xinlong Zhou^1^^#^, Haiming Yuan^1#^

1.Dongguan Maternal and Child Health Care Hospital. Dongguan 523120, China.

2. Dongguan Labway Clinical Laboratory Co., Ltd, Dongguan 523429, China

^#^Correspondence

Xinlong Zhou Email:149191818@qq.com

Haiming Yuan Email: [haimingyuan@sina.cn](mailto:haimingyuan@sina.cn)

**Protein structure data and analysis**

To investigate the effects of the c.4T>G p.Cys2Gly mutation on the ASNS protein, a comprehensive computational approach was employed. Initially, the amino acid sequence of the ASNS protein was retrieved from the UCSC Genome Browser website (http://genome.ucsc.edu/) based on the transcript NM_133436. The mutation site and the corresponding amino acid change were identified, and the mutant amino acid sequence was generated. Then, the physicochemical properties of both the wild-type and mutant ASNS proteins were analyzed using the ProtParam tool available on the ExPASy (https://web.expasy.org/protparam/). Additionally, the secondary structure predictions for the wild-type and mutant ASNS proteins were performed using the SOPMA program (https://web.expasy.org/protparam/). To obtain a structural model of the wild-type ASNS protein, the AlphaFold2 server (https://colab.research.google.com/github/sokrypton/ColabFold/blob/main/AlphaFold2.ipynb) was utilized. The modeling data were obtained, and the PDB file of the rank1 model was selected as the structure of the wild-type protein. The evolutionary conservation of the wild-type ASNS protein structure was analyzed using ConSurf (https://consurf.tau.ac.il/consurf_index.php). Site-directed mutagenesis and structural analysis were performed on the wild-type ASNS protein using the PyMOL Molecular Graphics System (Version 2.0). The Adaptive Poisson-Boltzmann Solver (APBS) module in the ChimeraX visualization software (https://www.cgl.ucsf.edu/chimerax/) was employed to generate electrostatic potential maps and analyze the changes in surface electrostatic potential before and after the mutation. The potential for structural disruption caused by the mutation was predicted using the Missense3D (http://missense3d.bc.ic.ac.uk/~missense3d/). The stability of the ASNS protein upon the c.4T>G p.Cys2Gly mutation was analyzed using several online prediction tools, including DUET (http://biosig.unimelb.edu.au/duet/stability), MUpro (https://mupro.proteomics.ics.uci.edu/), DynaMut2 (https://biosig.lab.uq.edu.au/dynamut2/submit_prediction_mm), SAAFEC-SEQ (http://compbio.clemson.edu/SAAFEC-SEQ/#started), and I-Mutant2.0 SEQ (http://compbio.clemson.edu/SAAFEC-SEQ/#started). Finally, ΔΔG (kcal/mol) value [Caswell et al., 2022] is derived, and its negative value shows that the stability of the protein is affected after the mutation.

Alphafold2 modeling and PyMOL structural mapping revealed that the p.Cys2Gly variant does not disrupt the tertiary structure of the ASNS protein (Figure S1). Consurf analysis identified Cys2 as a deeply buried residue with maximal structural conservation (score 9/9), indicating its essential role in maintaining protein architecture. Although this mutation preserves the local hydrogen-bonding network, computational predictions from five independent algorithms consistently demonstrated reduced protein stability (ΔΔG<0 kcal/mol, Figure S2). This destabilization likely arises through two non-hydrogen-bond-dependent mechanisms: (1) disruption of hydrophobic interactions in the protein core due to the replacement of cysteine's -CH2SH side chain with glycine's single hydrogen atom, and (2) introduction of excessive backbone flexibility characteristic of glycine, which possesses unrestricted φ/ψ dihedral angles. Glycine's unique structural properties - absence of a side chain and exceptional conformational freedom - may amplify these destabilizing effects at this evolutionarily conserved position. These findings collectively suggest that the mutation compromises protein folding via perturbations in hydrophobic packing and/or increased conformational entropy, rather than through direct structural disruption.


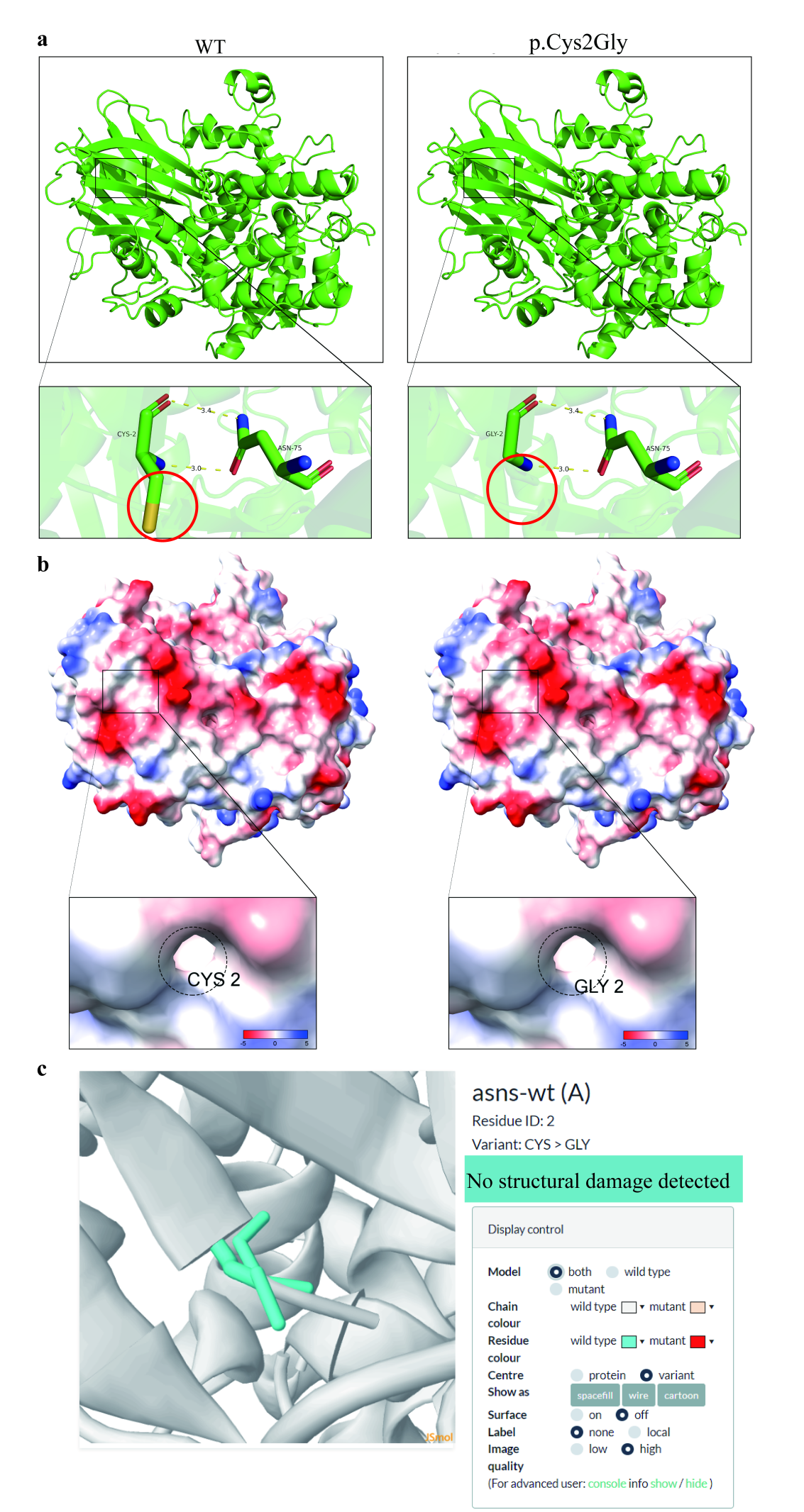


**Figure S1**. a. Effect of the p.Cys2Gly variant on tertiary structure. b. Effect of the p.Cys2Gly variant on protein surface potential. c. Prediction of destructive effect of the p.Cys2Gly variant on 3D structure.


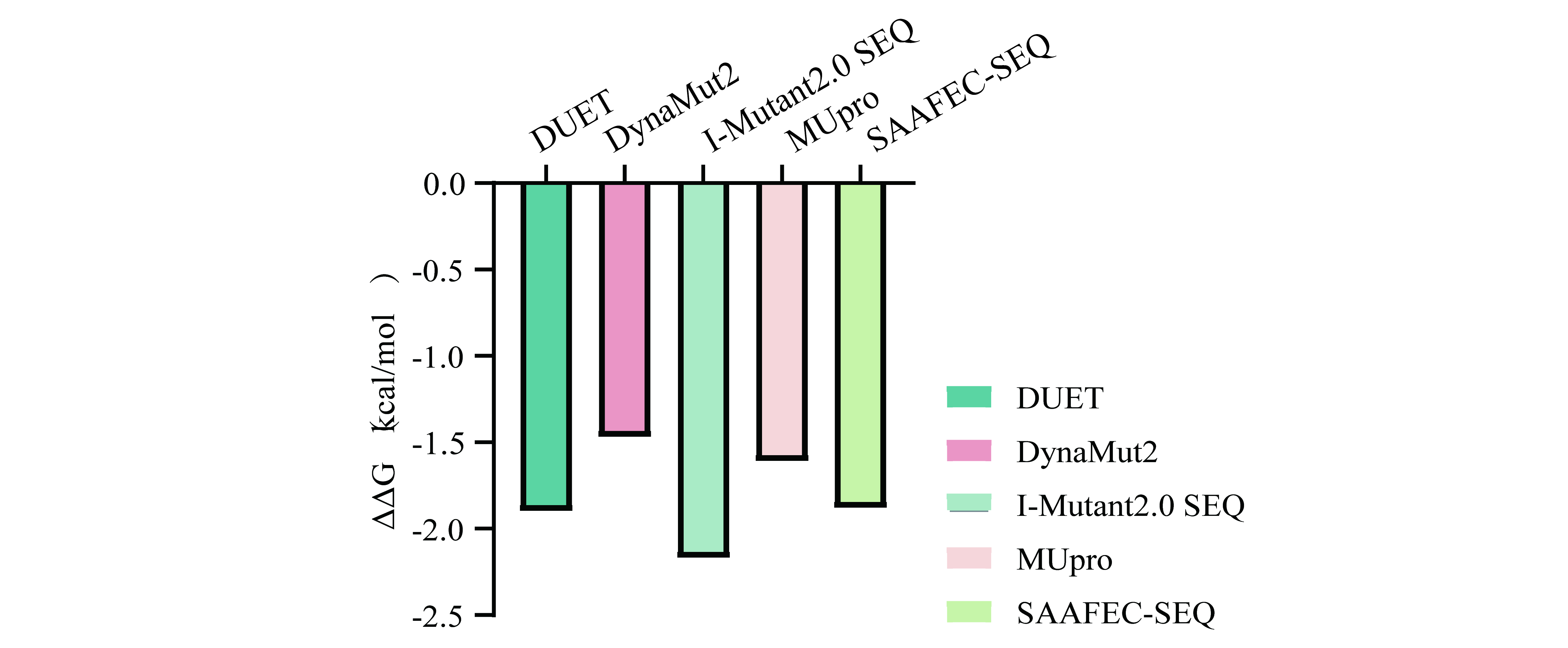


**Figure S2**. The c.4T>G (p.Cys2Gly) variant affects protein stability. The ΔΔG (kcal/mol) value was derived from the protein stability prediction analysis, and its negative value showed that the protein stability was affected after mutation

Reference

Caswell RC, Gunning AC, Owens MM, Ellard S, Wright CF. Assessing the clinical utility of protein structural analysis in genomic variant classification: experiences from a diagnostic laboratory. Genome Med. 2022;14(1): 77. doi: 10.1186/s13073-022-01082-2.
